# Providing support to psychiatric patients living in the community in Japan: patient needs and care providers perceptions

**DOI:** 10.1186/1752-4458-2-5

**Published:** 2008-05-29

**Authors:** Hiroaki Miyata, Hisateru Tachimori, Tadashi Takeshima

**Affiliations:** 1 Department of Healthcare Quality Assessment, Graduate School of Medicine, University of Tokyo, Japan; 2 Department of Mental Health Administration, National Institute of Mental Health, Japan

## Abstract

**Background:**

Social support programs are a critical component of care for psychiatric patients living in community or residential settings. There is little information, however, on how to optimally deliver these services in the Japanese context.

**Methods:**

We selected ten community life support centers for patients with major mental illness and administered questionnaires to 199 pairs of patients and staff members. These questionnaires consisted of twenty-six items from six categories: difficulties with interpersonal relationships; risks to physical well-being; risks to mental health; difficulties with life skills; challenges regarding living conditions; risks towards community safety. For each of these items, patients were asked whether they had experienced difficulties during the previous month, and staff members were asked the extent to which their patients needed support.

**Results:**

The results demonstrated that staff members tended to understate patients' needs regarding chronic medical conditions (p < 0.01), dietary habits (< 0.01), and excessive smoking or alcohol drinking (< 0.05). On the other hand, staff members recognized patients' needs regarding mental health problems to a greater extent than patients themselves (< 0.05).

**Conclusion:**

Results of this study suggest that social services geared towards specific tasks of daily living form an important component of comprehensive care for psychiatric patients living in community settings in Japan.

## Background

It is clear from real-world programs and clinical research that integrated community services form a critical component of care for individuals suffering from severe mental illness. Residence in closed hospital settings often leads to the institutionalization of the psychiatric patient, which further worsens motivation and living skills [1]. To the extent feasible for patient safety, psychiatric care should be provided in environments as minimally restrictive as possible, ideally in community settings. In contrast to other developed countries, which have seen the massive deinstitutionalization of psychiatric patients over the past thirty years, psychiatric care in Japan remains highly institutionalized [2,3]. This has been driven largely by private-sector psychiatric hospitals. These hospitals account for approximately 90% of psychiatric beds in Japan, and construction of new wards is ongoing on a large scale. This has led to Japan exhibiting the highest number of psychiatric beds per capita in the world, with 284 beds per 100,000 persons [4].

In September 2004, the Ministry of Health, Labor and Welfare, Japan announced a new policy aimed at expediting the discharge of 72,000 in-patients to community settings, contingent upon proper provision of adequate social support services. As a result of this initiative, it has become imperative to explore effective ways to provide these services in the Japanese context. Previous studies have suggested that approximately 30% of psychiatric patients currently in long-term institutional settings could lead safe and productive lives in the community [5,6]. Many patients, however, are at risk for poor adherence and relapse even if they exhibited strong adherence and therapeutic response in an in-patient setting [7]. Providing adequate social support services is crucial in reducing this risk. In Japan, however, few programs addressing these needs have been developed, and research into effective program design is lacking [8,9].

We have previously published the results of a pilot survey addressing social support services for psychiatric patients living in community settings in Japan [10]. In that study, we interviewed psychiatric patients, patients' families, care providers, and community residents about the challenges faced by psychiatric patients living in community settings. The framework of that study comprised challenges on the spectrum from daily frustrations to serious crises [11]. Here, we extend that research to a multi-center survey examining: i) difficulties with interpersonal relationships; ii) risks to physical well-being; iii) risks to mental health; iv) difficulties with life skills; v) challenges regarding living conditions; vi) risks towards community safety.

## Methods

### Participants

We selected ten social support centers for people with major mental illness in Japan. The social support centers provide counseling and offer advice for people with mental illness and their families. The centers also coordinate other social resources regarding mental health (i.e. public health centers, group homes, vocational training centers) and provide comprehensive livelihood support to them. The Japanese government sets standards for establishment of the social support centers and subsidizes their management. For each of these centers, we administered a single facility questionnaire and twenty individual questionnaires. We assigned one staff member to serve as data manager for each center. These individuals were responsible for distributing questionnaires and collecting completed forms. The facility questionnaire was administered to a single well-informed staff member at each center. For the individual questionnaires, we selected the first twenty registered patients who attended each the center after commencement of the study. Both the patients and the staff member were invited to fill complete these questionnaires.

### Questionnaires

Based on former research (content analysis toward over 1000 cases regarding risk and challenges in psychiatric patients' community life) [10] we identified six categories of risks and challenges as below.

A. Difficulties with interpersonal relationships (1. relationships with family and friends; 2. relationships with neighbors or other community members; 3. relationships with co-workers; 4. relationships with potential employers and colleagues)

B. Risks to physical well-being (5. dietary habits; 6. excessive smoking or alcohol drinking; 7. problems associated chronic medical illness or disability; 8. burden of support and care placed on the patient's family)

C. Risks to mental health (9. chronic mental health conditions; 10. ideation or actual self-injury; 11. ideation or actual violence towards others; 12. compliance with psychiatric medication and outpatient treatment; 13. mistrust of drug use and treatment policy)

D. Difficulties with life skills (14. use of public institutions; 15. management of household expenditures; 16. performance of community service (e.g. cleanup duty in the community) ; 17. execution of trash separation; 18. preparation of food)

E. Challenges regarding living conditions (19. adapting to changes in one's daily environment; 20. adapting to changes in residential environment; 21. practicing fire prevention; 22. cleaning one's room)

F. Risks towards community safety (23. property management; 24. attitudes toward at-home sales; 25. attitudes toward natural disasters; 26. attitudes toward crime)

Support centers were asked whether they have experienced difficulties in addressing these issues with their clients over the previous month. Similarly, patients were asked whether they received support in dealing with these issues over the past month. Staff members of the support centers were asked the extent to which their patients required support in each of the six domains over the previous month. Additionally, staff members were asked about the following patient characteristics: sex, age, diagnosis, state of residence, family, job status, hospital admission, duration of hospital stays, outpatient treatment. In the facility questionnaire, we asked about the management agency, the number of full-time staff, the number of part-time staff, and the number of registered patients over the previous month.

### Statistical analysis

We used basic descriptive statistics to describe the distributions of characteristics of the patient population, characteristics of the facilities, staff awareness regarding support needs of patients, patients' difficulty in dealing with risks and problems, and the degree to which support was helpful to users of the support centers.

To examine the relationship between patients' characteristics and their difficulties in dealing with risks and problems, we used Fisher exact test for categorical variables and the students' T test for continuous variables. In this analysis, patients' difficulties were divided into two categories (no experience or never; sometimes or often). Diagnosis (schizophrenia; other mental illness) and duration of hospital stays (under ten years; ten years and more years) were also divided into two categories.

We used the Wilcoxon signed-rank test to examine differences between patients' difficulties and staffs recognition of patients' support needs. Staff recognition of patients' support (unknown or none; sometimes or highly) and patients' difficulties (no experience or never; sometimes or highly) were again divided into two categories. All reported p values are two-sided. Statistical analyses were conducted using SPSS version 13.0J.

## Results

Facility questionnaires were completed at all participating facilities. Four facilities were managed by a social welfare corporation, three facilities by a medical corporation, one facility by a municipal corporation, and one facility by an incorporated nonprofit organization. Four facilities had three staff members, three facilities had four staff members and three facilities had five staff members; two facilities had no part-time staff members, six facilities had two part-time staff members, and two facilities had three part-time staff members. One facility had fewer than one hundred patients, six facilities had one hundred to two hundred patients, and three facilities had at least two hundred patients.

Individual questionnaires were obtained from 199 patients at the ten facilities. Characteristics of participants are shown in Table 1. Of the 199 participants, 136(68.3%) were male, and mean age was 44.0(SD 11.6, range 22–73). The primary psychiatric diagnosis was schizophrenia in 153(76.9%); of the others, 17(8.5%) patients had a mood disorder, 7(3.5%) suffered from psychoneurosis, 2(1.0%) had a substance-related disorder, and 2(1.0%) had a personality disorder. With regards to place of residence, 102(51.3%) lived in a house they owned themselves, 66(33.2%) lived in rental housing, 15(7.5%) lived in a group home, 7(3.5%) lived in life skills training accommodations, and 5(2.5%) lived in communal dwellings (which are much the same as group homes, but do not receive support from government).

**Table 1:** Characteristics of the study population (N = 199)

		Mean	SD	Range
Age		44.0-	11.6	22–73

			N	%

Sex	Male		136	68.3

Resident state	Home owner		102	51.3
	Rental housing		66	33.2
	Group home		15	7.5
	Communal dwelling		5	2.5
	Skills training accommodations		7	3.5
	Other		4	2.0

Job Status	Unemployed		114	57.3
	Full-time		4	2.0
	Part-time		20	10.1
	Vocational aid center		44	22.1
	Other		17	8.5

Diagnosis	Schizophrenia		153	76.9
	Mood disorder		17	8.5
	Substance-related disorder		2	1.0
	Psychoneurosis		7	3.5
	Personality disorder		2	1.0
	Other		18	9.0

Experience of admission	Never		27	13.6
	In a year		25	12.6
	More than a year ago		143	71.9

Total duration of hospital stay	Zero		27	13.6
	Less than 1 year		47	23.6
	1 – 10 years		100	50.3
	10 – 20 years		14	7.0
	20 years and more		3	1.5

Staff perceptions regarding the support needs of patients are presented in Table 2. Most staff members thought that patients required support to improve relationships with family and friends, to obtain a job, to deal with mental health problems, and to improve relationships with co-workers. On the other hand, only a few staff members thought that patients needed support in practicing fire prevention, in improving attitudes toward crime and violence, or in using public institutions.

**Table 2:** Staff awareness regarding the social support needs of patients (N = 199)

		**Highly**	**sometimes**	**none**	**unknown**
Interpersonal Relationships & Life Skills	use of public institution	4.0%	15.1%	69.3%	11.6%
	management of household expenditure	9.0%	21.1%	60.3%	9.5%
	performance of community service	3.5%	17.6%	48.7%	30.2%
	execution of trash separation	4.5%	19.1%	60.3%	16.1%
	preparation of food	9.5%	38.7%	42.2%	9.5%
	relationship with potential employers and colleagues	18.6%	42.7%	32.2%	6.5%
	relationship with family and friends	11.1%	56.8%	24.6%	7.5%
	relationship with neighbors or other community members	5.5%	34.2%	36.7%	23.6%
	relationship with co-workers	9.5%	45.2%	35.2%	10.1%

Living Conditions	adapting to changes in one's daily environment	14.1%	32.2%	30.7%	23.1%
	adapting to changes in residential environment	8.0%	24.1%	47.7%	20.1%
	practicing fire prevention	2.5%	14.1%	73.9%	9.5%
	cleaning one's room	6.0%	17.6%	60.8%	15.6%

Physical Well-being	dietary habits	8.5%	35.2%	46.2%	10.1%
	excessive smoking or alcohol drinking	4.5%	24.1%	61.8%	9.5%
	problems associated chronic medical illness or disability	9.5%	39.7%	41.7%	9.0%
	burden of support and care placed on the patient's family	6.5%	30.2%	45.7%	17.6%

Mental Health	chronic mental health conditions	8.0%	50.8%	32.2%	9.0%
	ideation or actual self-injury	4.5%	18.6%	63.8%	13.1%
	ideation or actual violence towards others	4.5%	14.1%	67.3%	14.1%
	compliance with psychiatric medication and outpatient treatment	5.5%	19.6%	69.8%	5.0%
	mistrust of drug use and treatment policy	3.5%	33.7%	55.8%	7.0%

Community safety	property management	9.5%	18.1%	49.2%	23.1%
	attitude toward at-home sales	3.5%	22.1%	55.8%	18.6%
	attitude toward natural disasters	4.0%	27.6%	52.3%	16.1%
	attitude toward crimes	1.0%	16.6%	65.8%	16.6%

Patients' difficulties in dealing with risks and challenges are presented in Table 3. Most patients experienced difficulty in dealing with relationships with family and friends, in dealing with chronic illness, in improving dietary habits, and in managing household expenditure. On the other hand, few patients found difficulty in performing community service, avoiding self-injury or violence, maintaining positive attitudes towards at-home sales, or using public institutions.

**Table 3:** Patients' difficulties in addressing risks and challenges (N = 199)

		**highly**	**some times**	**never**
Interpersonal Relationships & Life Skills	use of public institution	8.8%	27.7%	63.5%
	management of household expenditure	20.2%	42.6%	37.2%
	performance of community service	16.9%	34.8%	48.3%
	execution of trash separation	11.2%	39.6%	49.1%
	preparation of food	21.3%	43.1%	35.6%
	relationship with potential employers and colleagues	23.2%	41.5%	35.4%
	relationship with family and friends	25.3%	51.1%	23.7%
	relationship with neighbors or other community members	17.1%	46.1%	36.8%
	relationship with co-workers	21.6%	43.9%	34.5%

Living Conditions	adapting to changes in one's daily environment	32.4%	45.1%	22.5%
	adapting to changes in residential environment	18.1%	45.7%	36.2%
	practicing fire prevention	11.9%	25.0%	63.1%
	cleaning one's room	14.3%	40.5%	45.2%

Physical Well-being	dietary habits	25.1%	44.0%	30.9%
	excessive smoking or alcohol drinking	28.9%	40.6%	30.5%
	problems associated chronic medical illness or disability	29.9%	48.2%	22.0%
	burden of support and care placed on the patient's family	17.8%	48.1%	34.1%

Mental Health	chronic mental health conditions	22.8%	35.3%	41.9%
	ideation or actual self-injury	25.4%	23.8%	50.8%
	ideation or actual violence towards others	13.9%	27.0%	59.0%
	compliance with psychiatric medication and outpatient treatment	14.0%	24.7%	61.3%
	mistrust of drug use and treatment policy	11.0%	26.5%	62.4%

Community safety	property management	23.4%	26.6%	50.0%
	attitude toward at-home sales	14.8%	38.0%	46.3%
	attitude toward natural disasters	13.7%	41.9%	44.4%
	attitude toward crimes	8.6%	42.2%	49.1%

The social support services used by patients are shown in table 4. Table 5 depicts the extent to which these services were perceived as helpful for patients in dealing with the challenges they were facing. Many patients thought that the support was useful in managing household expenditures, maintaining adherence to medication and outpatient treatment, preparing food, and improving relationships with coworkers. On the other hand, few patients thought the support was useful in improving attitudes towards crime, decreasing smoking or alcohol drinking, improving attitudes toward at-home sales, or establishing better relationships with neighbors.

**Table 4:** Social support services received by patients (N = 199)

		**received support**
Interpersonal Relationships & Life Skills	use of public institution	35.7%
	management of household expenditure	45.2%
	performance of community service	26.1%
	execution of trash separation	35.2%
	preparation of food	52.8%
	relationship with potential employers and colleagues	58.3%
	relationship with family and friends	61.3%
	relationship with neighbors or other community members	36.2%
	relationship with co-workers	51.3%

Living Conditions	adapting to changes in one's daily environment	39.2%
	adapting to changes in residential environment	32.2%
	practicing fire prevention	25.6%
	cleaning one's room	37.7%

Physical Well-being	dietary habits	44.2%
	excessive smoking or alcohol drinking	29.6%
	problems associated chronic medical illness or disability	61.8%
	burden of support and care placed on the patient's family	41.7%

Mental Health	chronic mental health conditions	53.3%
	ideation or actual self-injury	32.2%
	ideation or actual violence towards others	31.2%
	compliance with psychiatric medication and outpatient treatment	45.2%
	mistrust of drug use and treatment policy	47.7%

Community safety	property management	35.2%
	attitude toward at-home sales	22.6%
	attitude toward natural disasters	26.6%
	attitude toward crimes	25.1%

**Table 5:** Degree to which patients viewed support services as helpful at addressing their risks and challenges

		**very helpful**	**helpful**	**not so helpful**
Interpersonal Relationships & Life Skills	use of public institution	54.9%	31.0%	14.1%
	management of household expenditure	52.2%	37.8%	10.0%
	performance of community service	26.9%	42.3%	30.8%
	execution of trash separation	31.4%	40.0%	28.6%
	preparation of food	52.4%	35.2%	12.4%
	relationship with potential employers and colleagues	42.2%	42.2%	15.5%
	relationship with family and friends	44.3%	38.5%	17.2%
	relationship with neighbors or other community members	26.4%	37.5%	36.1%
	relationship with co-workers	42.2%	45.1%	12.7%

Living Conditions	adapting to changes in one's daily environment	39.7%	42.3%	17.9%
	adapting to changes in residential environment	34.4%	42.2%	23.4%
	practicing fire prevention	29.4%	43.1%	27.5%
	cleaning one's room	34.7%	38.7%	26.7%

Physical Well-being	dietary habits	36.4%	38.6%	25.0%
	excessive smoking or alcohol drinking	18.6%	37.3%	44.1%
	problems associated chronic medical illness or disability	37.4%	45.5%	17.1%
	burden of support and care placed on the patient's family	36.1%	44.6%	19.3%

Mental Health	chronic mental health conditions	36.8%	45.3%	17.9%
	ideation or actual self-injury	26.6%	40.6%	32.8%
	ideation or actual violence towards others	27.4%	46.8%	25.8%
	compliance with psychiatric medication and outpatient treatment	45.6%	43.3%	11.1%
	mistrust of drug use and treatment policy	34.7%	46.3%	18.9%

Community safety	property management	44.3%	34.3%	21.4%
	attitude toward at-home sales	22.2%	40.0%	37.8%
	attitude toward natural disasters	30.2%	41.5%	28.3%
	attitude toward crimes	24.0%	28.0%	48.0%

Older patients experienced significantly more difficulty in preparing food (p < 0.05) and at having positive attitudes towards at-home sales (p < 0.05). Younger patients experienced significantly more difficulty in obtaining a job (p < 0.01), avoiding self-injury (p < 0.01), and restraining themselves from violence towards others (p < 0.01). Men reported greater difficulty in refraining from excessive smoking or alcohol drinking (p < 0.05). Patients living alone reported greater difficulty in cleaning one's own room (p < 0.01), preparing food (p < 0.01), and separating trash (p < 0.05) than those living with families. Patients suffering from schizophrenia experienced more difficulty in practicing appropriate fire prevention (p < 0.05) than patients with other mental disorders. Finally, patients whose duration of hospital stays were ten or more years reported greater difficulty in preparing food (p < 0.05), cleaning one's own room (p < 0.01), and adhering to medication and outpatient treatment regimens (p < 0.05).

Table 6 presents the differences between patients' reported difficulties and staff members' perceptions of patients' support needs. Staff perception of patients' support needs were significantly higher for mental health problems (p < 0.05) than patients' reported difficulties. Staff perceptions of support needs were significantly lower than those of patients' reported difficulties on the following ten items: using public institutions (p < 0.05), managing household expenditures (p < 0.01), separating trash (p < 0.01), practicing appropriate fire prevention (p < 0.01), cleaning one's room (p < 0.01), preparing food (p < 0.01), abstaining from excessive smoking or alcohol drinking (p < 0.05), dealing with chronic medical illness (p < 0.01), adhering to medication and outpatient treatment regimens (p < 0.05), and refraining from violence towards others (p < 0.01).

**Table 6:** Differences between staff perception of patients' support needs and their difficulties (N = 199)

		**staffs' recognition of support needs**	**users' difficulty**
Interpersonal Relationships & Life Skills	use of public institution	19.1%	29.1% *
	management of household expenditure	30.2%	57.8%* *
	performance of community service	21.1%	23.1%
	execution of trash separation	23.6%	43.2%* *
	preparation of food	48.2%	51.8%
	relationship with potential employers and colleagues	61.3%	53.3%
	relationship with family and friends	67.8%	71.4%
	relationship with neighbors or other community members	39.7%	48.2%
	relationship with co-workers	54.8%	48.7%

Living Conditions	adapting to changes in one's daily environment	46.2%	39.7%
	adapting to changes in residential environment	32.2%	30.2%
	practicing fire prevention	16.6%	29.6%* *
	cleaning one's room	23.6%	46.2%* *

Physical Well-being	dietary habits	43.7%	60.8%* *
	excessive smoking or alcohol drinking	28.6%	44.7%* *
	problems associated chronic medical illness or disability	49.2%	64.3%* *
	burden of support and care placed on the patient's family	36.7%	42.7%

Mental Health	chronic mental health conditions	58.8% *	48.7%
	ideation or actual self-injury	23.1%	32.2%
	ideation or actual violence towards others	18.6%	25.1%
	compliance with psychiatric medication and outpatient treatment	25.1%	36.2%*
	mistrust of drug use and treatment policy	37.2%	34.2%

Community safety	property management	27.6%	32.2%
	attitude toward at-home sales	25.6%	28.6%
	attitude toward natural disasters	31.7%	34.7%
	attitude toward crimes	17.6%	29.6%* *

*p < 0.05

** p < 0.01

## Discussion

We identified 25 risks and challenges from previous research in Japan [10] and these items were almost identical with former studies in western country (CANSAS-P) [12] with a few exceptions. There were no items regarding "performance of community service" and "attitudes toward at-home sales" in CANSAS-P. This might be due to difference of style of community life between Japan and western countries (i.e. there is cleanup duty in community and sales person frequently visit one's home.)

Fostering effective and healthy interpersonal relationships is thought to be a critical component for improving the quality of community life of psychiatric patients in Japan. In our study, however, approximately 70% of patients reported experiencing difficulty in maintaining healthy interpersonal relationships with family and friends. Moreover, a large number of patients reported difficulty in other items requiring strong interpersonal skills. These included looking for a job (64.7%), interacting productively with coworkers (65.5%), and relating with neighbors (63.2%). Meanwhile, a large number of support staff perceived their patients as having difficulty with maintaining interpersonal relationships with family and friends (67.9%). Other items regarding interpersonal relationships were also listed at the top of staff members' perceptions of patient needs. As Killaspy [13] suggested trusting and stimulating relationship between clients and professionals were most important. In this study both patients (71.4%) and staff members (67.8%) shared the belief that interpersonal relationships were important issues in patients' ability to adapt to community life. Patients did use support services to address these difficulties, particularly support with interpersonal relationships: 61% of patients received support for relationships with family and friends, 58% received support for obtaining a job, 51% received aid in improving relationships with coworkers, and 36% received support in improving relationship among neighbors. Despite having received this support, few patients (26.4%) thought that the support oriented towards relationships among neighbors was helpful. As such, our data suggest the need for new community interventions aimed at improving interpersonal relationships among these patients.

Many patients also reported difficulties in dealing with chronic medical conditions (78.1%) or in improving dietary habits (69.1%) and in abstaining from excessive smoking or drinking (69.5%). Though Crawford et al [14] suggested that both service users and providers have reported that delivering continuity of care to people with severe mental illness should be a service priority, the staff members we interviewed failed to perceive these as significant needs in their patients. In improving dietary habits, it appears that patients require more support (60.8% users' difficulty, 43.7% staff's recognition of support needs) in education and adherence to the dietary regimen rather than support in food preparation per se (51.8% user's difficulty, 48.2% staff's recognition of support needs). In decreasing cigarette and alcohol consumption, new strategies are needed because few of the patients (18.6%) surveyed felt the support services they were receiving were working.

Many community center patients found it difficult to deal with mental health risks because patients tended to report these as being lower-priority needs. For example, dealing with mental health conditions (7^th ^of 26 needs), avoiding self-injury (18^th ^of 26), restraining from violence towards others (25^th ^of 26), adhering to medication and outpatient treatment regimens (22^nd ^of 26), and abstaining from illicit drugs (24^th ^of 26) were listed as low-priority by many patients. Staff members tended to recognize these as problems to a greater extent than the patients themselves. The reason for this may be that, on the one hand, staff members worry about mental health problems of their patients, while on the other, patients may lack awareness about their own mental health issues.

The major challenge identified by patients in the area of life skills was in the management of household expenditures (62.8%). Additionally, over half of the patients reported difficulty in food preparation (64.4%), in cleaning one's room (54.8%), or in trash separation (50.8%), while fewer than 40% of patients reported difficulty in managing other risks and problems. Staff members did not fully appreciate the extent to which these issues were challenging their patients. While 58% of patients reported difficulties in managing household expenditures, only 30% of staff members thought their patients needed support in this domain. Similarly, patients' needs were reported at significantly higher level than staff perceptions in the domains of using public institutions (user's difficulty 29.1%, staff's recognition of support needs 19.1%), trash separation (user's difficulty 43.2%, staff's recognition of support needs 23.6%), practicing fire prevention (user's difficulty 57.8%, staff's recognition of support needs 30.2%), and cleaning one's own room (user's difficulty 46.2%, staff's recognition of support needs 23.6%). These results suggest that patients with mental disorders living in the community require practical support related to performing the tasks of daily life in addition to the conventional support focused on mental health issues specifically. Few patients were satisfied with the support they were receiving through the community centers. As previous studies have suggested, unmet needs are a strong predictor of less favorable health perceptions and a lower quality of life [15,16], Addressing this disconnect between patient needs and service provision is important. It will be necessary to enhance linkages and collaboration between community center staff and other mental health and social service professionals in Japan.

There were several limitations of this study. Participants of this study did not live exclusively in the community, and they all utilized community support center services. It is likely that this study underrepresented the most highly functioning patients as well as the most reclusive or lowest functioning patients, as both may be less likely to utilize community support center services. Additionally, our sampling strategy was not randomized; for purposes of convenience and to decrease recall bias, we only surveyed the first twenty registered patients who attended each centre following the commencement of the study. Though we selected 10 community support centers in consideration of characteristics diversity, generalizability of the study findings is limited because of the small sample size. Mitigating these limitations, however, was the fact that none of the patients or staff members who were approached declined to participate, and the number of missing values in each item was insignificant.

## Conclusion

Results of this study suggest that social services geared towards specific tasks of daily living form an important component of comprehensive care for psychiatric patients living in community settings in Japan.

## Competing interests

The authors declare that they have no competing interests.

## Authors' contributions

HM and HT originated the project and develop questionnaire, HM wrote the manuscript, HT and TT reviewed the manuscript. All authors read and approved the final manuscript.

## References

[B1] Wing JK, Brown GW (1970). Institutionalism and Schizophrenia.

[B2] Knudsen H, Thornicroft G (1996). Mental health service evaluation.

[B3] Oshima I, Mino Y, Inomata Y (2003). Institutionalisation and schizophrenia in Japan: social environments and negative symptoms. Nationwide survey of in-patients. Br J Psychiatry.

[B4] Horiuchi K, Nishio M, Oshima I, Ito J, Matsuoka H, Tsukada K (2006). The quality of life among persons with severe mental illness enrolled in an assertive community treatment program in Japan: 1-year follow-up and analysis. Clinical practice and Epidemiology in Mental Health.

[B5] Kuroda K, Toida S, Kawamuro Y (1999). Possibility of discharge and need for rehabilitation of psychiatric patients hospitalized for one year or more in Japan: a preliminary report. Committee on Rehabilitation Affairs, Subcommittee of Survey on Rehabilitation Need. Seishin Shinkeigaku Zasshi.

[B6] Oshima I, Inomata Y, Toida S, Yoshizumi A, Inachi S, Maruyama S (1991). Possibility to be discharged of long-stay psychiatric patients and their suitable accommodation need in Japan: nationwide survey of 40000 psychiatric beds. Seishin Shinkeigaku Zasshi.

[B7] Yamada K, Watanabe S, Yagi G (1999). Analysis and evaluation of factors associated with noncompliance. Rinsho Seishin Igaku.

[B8] Hata T, Aso Y, Akiyama N, Kaneko M (2003). Psychiatric patients' needs and uses of resource: results from patient's family survey. Seishin Igaku.

[B9] Suzuki Y, Kitano K, Haruhara M, Yanagisawa S, Sasaki R, Hatayama Y (2002). Improving community life of psychiatric patients' support: From the local point of view. Kousyu Eisei.

[B10] Tachimori H (2004). Improving psychiatric emergency system from the view point of patients and families: A qualitative study.

[B11] Watanabe T, Sugawara M, Shibata Y (2001). Factor analysis regarding interruption of community life in group home for mental health disorder. Nihon syakai seishin igaku zasshi.

[B12] Trauer T, Tobias G, Slade M (2007). Development and evaluation of a patient-rated version of the camberwell assessment of need short appraisal schedule (CANSAS-P). Community mental health journal.

[B13] Killaspy H (2006). From the asylum to community care: learning from experience. British Medical bulletin.

[B14] Crawford MJ, Jonge E, Freeman GK, Weaver T (2004). Providing continuity of care for people with severe mental illness. Soc Psychiatry Psychiatr Epidemiol.

[B15] Wiersma D (2006). Needs of people with severe mental illness. Acta Psychiatr Scand.

[B16] Slade M, Leese M, Cahill S, Thornicroft G, Kuipers E (2005). Patient-rated mental health needs and quality of life improvement. British Journal of Psychiatry.

